# Trientine ameliorates bleomycin-induced pulmonary fibrosis in rats through copper chelation and modulation of the CTR1/LOX/COL pathway

**DOI:** 10.1007/s10787-026-02135-3

**Published:** 2026-03-05

**Authors:** Ibrahim T. Khalil, Mahmoud Elshal, Norhan M. Elsayed, Asmaa Radwan

**Affiliations:** 1 Department of Research and Development, Dawzan Pharmaceutical Company, Cairo, Egypt; 2 Department of Pharmacology and Toxicology, Faculty of Pharmacy, Mansoura University, Mansoura, Egypt; 3 AlGhad College for Applied Medical Sciences, Najran, Saudi Arabia; 4 Department of Pharmacology & Toxicology, Faculty of Pharmacy, Suez Canal University, Ismailia, 41522 Egypt

**Keywords:** Pulmonary fibrosis, Trientine, Copper transporter, LOX enzymes, Drug repurposing

## Abstract

Pulmonary fibrosis (PF) remains a devastating disease. Copper (Cu), an essential trace element, has been implicated in fibrotic processes in various organs, including the lungs. This study investigated the potential protective effects of trientine (TRI), a copper chelator, against bleomycin (BLM)-induced PF in rats and explored the underlying mechanisms. PF was induced by intratracheal administration of BLM (5 mg/kg). Rats were randomly allocated into seven groups: normal control, TRI alone, BLM control, and four BLM plus TRI prophylactic or therapeutic groups receiving low (55 mg/kg) or high (110 mg/kg) TRI doses orally. Results demonstrated that TRI dramatically reversed BLM-elevated serum levels of lactate dehydrogenase and alkaline phosphatase, markers of tissue damage. The protective effects of TRI were multifaceted: it exhibited potent antifibrotic activity by reducing collagen buildup and inhibiting TGF-β, CTR1, LOX, and COL1 expression; it acted as an antioxidant by increasing antioxidant status and significantly upregulating Nrf2; and it served as an anti-inflammatory agent by decreasing inflammatory cytokines (TNF-α and IL-6) and modulating the NF-κB pathway by increasing NF-κB expression while limiting its activation and nuclear translocation, thereby reducing inflammatory signaling. Histopathological and immunohistochemical analyses confirmed substantial attenuation of collagen deposition and structural lung damage. Overall, TRI demonstrated potent antifibrotic, antioxidant, and anti-inflammatory properties, effectively protecting lung tissue against BLM-induced injury. These findings highlight copper chelation as a promising therapeutic strategy for pulmonary fibrosis and warrant further investigation into TRI’s clinical potential.

## Highlights

- Trientine attenuates bleomycin-induced pulmonary fibrosis in rats.
- Copper chelation reduces tissue injury and fibrotic marker expression.
- Trientine enhances antioxidant defenses via Nrf2 activation.
- Anti-inflammatory effects achieved by modulating NF-κB and lowering cytokines.
- Histology confirms reduced lung damage and collagen deposition.

## Introduction

Pulmonary fibrosis (PF) is a progressive complex lung disorder characterized by excessive pathological extracellular matrix (ECM) deposition, in the form of proteins like collagen (Todd et al. 2012; Valenzuela and Cottin 2022). PF has with few choices for treatment and a high death rate, however nintedanib and pirfenidone are two medications that have been approved for its treatment (Glass et al. 2020). Tyrosine kinase inhibitors like nintedanib and oral pyridines like pirfenidone have anti-inflammatory, antioxidant, and anti-fibrotic properties. Although the two medications have been shown to reduce the decline in lung function and halt the advancement of the disease, they have also been linked to certain adverse effects and concerns with acceptability (Bonella et al. 2023). Therefore there is an urgent need for alternative treatment options that ensure efficacy and safety to protect against such progressive lung disease.

Copper (Cu) is an essential element which is an important auxiliary component of many metabolic processes, such as mitochondrial respiration, redox reactions, and enzyme catalysis (Ruiz et al. 2021). Normally, the amount of Cu in cells is strictly controlled and kept at a relatively low level; too much cu can cause cytotoxicity or even cause cell death (Lutsenko et al. 2025). Recently different evidence indicate that fibrosis in a variety of organs, including the human liver (Padrilah et al. 2017), the human oral submucous tissue (Yadav et al. 2015), the rat kidney (Niu et al. 2020), the mouse salivary gland (Nam et al. 2024), human (Bargagli et al. 2017) and the animal lung (Li et al. 2022) are linked to Cu buildup.

In mammals, the copper transporter CTR1 is principally responsible for cellular Cu uptake. According to previous research, Cu ions can flow through the lipid bilayer when CTR1 forms a homotrimer with a minimal membrane spanning pore of 9 Å (De Feo et al. 2009; Wang et al. 2011). Numerous disorders, including immunological function, neurological illnesses, cardiovascular health, cancer, fertility, and reproductive health, were clearly associated with increased expression of CTR1 (Kim et al. 2013).

Lysyl Oxidase (LOX) enzymes are a group of extracellular Cu-dependent enzymes (Zaffryar-Eilot and Hasson 2022). They catalyze the formation of aldehydes from lysine residues in collagen and elastin precursors. These aldehydes react with unmodified lysine residues to form cross-links between collagen and elastin, which are critical for the integrity and elasticity of mature elastin as well as the stabilization of collagen fibrils (Eyre et al. 2019; Aronoff et al. 2021; Wang et al. 2021). LOX activity has been demonstrated to play a role in the pathogenesis of fibrotic disorders (Chen et al. 2018; Nguyen et al. 2020). These enzymes increase the cross-linking activity of insoluble extracellular proteins that are resistant to proteolysis. More specifically, dysregulated LOX activity leads to excessive or ineffective collagen cross-linking, which is the cause of numerous fibrotic diseases (Tjin et al. 2017; Laczko and Csiszar 2020; Pehrsson et al. 2021). The inhibition of the collagen cross-linking process makes sense to concentrate on creating anti-fibrotic treatments (Ovet and Oztay 2014; Chen et al. 2018).

Orphalan, Inc. produced trientine tetrahydrochloride (TRI, Cuvrior®), a copper chelator having triethylenetetramine (trientine) as its active component. It was appoved by FDA in 2022 for treatment of Wilson’s disease with a dual mechanism of action (Kamlin et al. 2024). TRI works systemically by removing absorbed Cu from the body by creating a stable complex that is subsequently excreyted through the urine. Further to its systemic action, TRI directly inhibits absorption of Cu from intestine, which lowers Cu uptake in the liver and transit in the portal vein (Kirk et al. 2024). Limited researches were established to study the effect of TRI on hypertrophic cardiomyopathy (Reid et al. 2022) and heart failure (Januzzi et al. 2024). It is the first time to study the effect of TRI in PF.

The BLM-induced PF model in rats is currently the most well-characterized and commonly used animal model because of its ease of induction, strong repeatability, and capacity to replicate most features of PF (Liu et al. 2017).

Accordingly, the present study aimed to investigate the possible beneficial effect of TRI on PF induced by BLM in rats and explore its underlying mechanisms, focusing on the potential role of CTR1 and LOX.

## Materials and methods

### Drugs and chemicals

Trientine tetrahydrochloride (CUVRIOR™, 300 mg/tablet) was provided by Orphalan (Paris, France); bleomycin (Bleocin, 15 I.U./vial) was provided by Nippon Kayaku Co. (Ltd., Tokyo, Japan); Thiopental sodium (Thiopental 500 mg/vial) was provided by Sandoz (Basel, Switzerland). Sodium Fucidate (fucidin, cream) was provided by LEO (Ballerup, Denmark).

### Experimental animals

Male adult Sprague Dawley rats weighing between 180 and 220 gm were acquired from the animal house of Suez Canal University, Faculty of Pharmacy. In individual cages, Rats were kept at room temperature (25 ± 2 °C), with a light–dark cycle of 12 h and a humidity of 60 ± 10%. Water was available to the rats at all times. Animals were fed a low-copper diet (Harlan Teklad, Madison, WI) with a copper content of approximately 2 mg/kg for 28 days prior to experimental procedures (Ovet and Oztay 2014). The experimental protocol was reviewed and approved by the Research Ethics Committee at the Faculty of Pharmacy, Suez Canal University, Egypt (approval code: 202305 MA2).

### Induction of pulmonary fibrosis

PF in rats was provoked by intratracheal injection of BLM at a dose of (5 mg/kg) dissolved in 0.1 ml of 0.9% normal saline (Borzone et al. 2001). Under thiopental sodium anesthesia (20 mg/kg body weight, i.p.), a midline neck incision was made to expose the trachea, and BLM was administered. To guarantee that BLM was distributed uniformly throughout the lung tissues, the rats were maintained in an upright posture, gently rubbed and turned many times. A 2% sodium fusidate cream was administered topically to the incision after it was surgically sutured (Zaghloul et al. 2017).

### Grouping of animals

Seven experimental groups of eight rats each were randomly assigned in the following way: Normal control group (Normal): rats were given 0.1 ml of normal saline intratracheally as previously described. Trientine group (TRI): rats received Trientine (110 mg/kg/day, oral) dissolved in 0.9% normal saline daily. Bleomycin control group (BLM): rats received bleomycin intratracheally at a dose of 5 mg/kg as previously described. BLM + TRI-L (P) and BLM + TRI-H (P): Two prophylacted groups which are pre-treated with two dose leves of TRI at 55 and 110 mg/kg day (Yin et al. 2016), 3 days before BLM administration and continued for 4 weeks after BLM administration. BLM + TRI-L (T) and BLM + TRI-H (T): Two treated groups which received TRI at 55 and 110 mg/kg day, on day eight after BLM administration and continued for 4 weeks.

At the end of the experiment, rats were given thiopental sodium (20 mg/kg body weight, i.p.) to induce anesthesia (Wada et al. 1996), then rats were euthanized. The retroorbital venous plexus was punctured to obtain blood samples, which were left to coagulate then centrifuged for 10 mintues at 4000 rpm to obtain sera that stored at -70°C for biochemical analyses (Parasuraman et al 2015). The lungs were removed, washed in ice-cold saline, and then divided into two parts, the first part was kept in formalin (4%) for histopathological and immunohistochemical analyses. Otherwise, the second part was further cut into 3 parts for subsequent homogenate preparation, Western blot analysis, and RT-PCR. Homogenization was done in PBS (pH 7.4) to produce a 10% w/v lung homogenate, which was centrifuged at 2000 rpm for 15 min at 4 °C. The supernatant was then separated (Molnar et al. 2021).

### Obtaining of bronchoalveolar lavage fluid (BALF)

The tracheas were exposed and cannulated, and then 3 ml of sterile 0.9% normal saline was gradually injected into the lungs three times, 1 ml at a time, through an opening in the thoracic cavity. This lavage volume (3 mL in three 1 mL aliquots) was specifically chosen based on previous rat studies and pilot trials to optimize sample recovery and minimize airway collapse, as recommended for rats weighing approximately 200–250 g. After repeatedly gently squeezing the chest, 50–70% of the recovery was obtained. Using a cooling centrifuge, BALF was centrifuged for 10 min at 2000 rpm and 4℃. To measure the amount of inflammatory cells and the total and differential cell counts, the sedimentation cell pellets were combined and re-suspended in sterile saline (Song e al. 2010).

### Determination of total protein content and lactate dehydrogenase activity in the BALF

The total protein content and LDH activity in the BALF were measured using commercial kits (Thermo Scientific, Rockford, USA; Human Diagnostics, Wiesbaden, Germany, respectively) following the manufacturer’s instructions. LDH was assessed as enzymatic activity in BALF to reflect local lung epithelial damage and cytotoxicity, where enzyme activity is a more relevant indicator of tissue integrity.

### Determination of lactate dehydrogenase, alkaline phosphatase, and serum ceruloplasmin activities

Serum LDH was measured by using commercial kit (Spinreact, S.A/S.A.U. Ctra. Santa Coloma, Spain). Serum LDH was expressed as concentration to indicate systemic tissue injury, which is standard practice for serum-based biochemical markers. The use of separate kits for BALF and serum was necessary because these matrices differ significantly in composition, and each kit is optimized for its respective sample type to ensure accuracy and sensitivity. ALP was measured by using commercial kit (Human Diagnostics, Wiesbaden, Germany). Ceruloplasmin was measured by using commercial kit (Accurex Biomedical Pvt. Ltd., Mumbai, India) following the manufacturer’s instructions.

### Evaluation of lung oxidative stress

Using commercially available assay kits (Biodiagnostic, Giza, Egypt), the lipid peroxidation biomarker MDA (CAT NO: MD 2529) as well as GSH (CAT NO: GR 2511) and SOD (CAT NO: SD 2521) were determined in the lung homogenate, as directed by the manufacturer.

### Histopathological examination

The left lungs were sectioned into 4-µm pieces after being cleaned and preserved in paraffin. Standard hematoxylin & eosin (H&E) and Masson’s trichrome staining techniques were applied. The inflammatory-cell infiltration was evaluated using the following scores: 0, no inflammation; 1, very mild; 2, mild; 3, moderate; 4, severe; and 5, intense inflammation (El-Kashef et al. 2022). The degree of fibrotic alterations in lung tissues challenged with BLM is assessed using a standardized quantification approach of PF in histological samples, as previously reported (Hübner et al. 2008).

### Immunohistochemical analysis of nuclear factor kappa B (NF-κB) and the nuclear factor erythroid 2-related factor 2 (Nrf2)

Rats’ lungs were dissected and preserved for 24 h in neutral buffered formalin. The materials were cleaned with xylene, embedded in paraffin wax, and dehydrated in increasing alcohol grades. Sections of the embedded materials, each 5 μm thick, were cut using a microtome. A light microscope (Olympus CH2, Japan) was used to examine the segment after it had been stained with hematoxylin and eosin.

Percentage expression of NRF2 and NF-κB in pulmonary tissue was determined in accordance with the guidelines provided by the relevant manufacturer. NRF2 analyzed at dilution of 1:1000 (from Servicebio, 22 Building, Biopark, Gaoxin 2nd Road No. 388, East Lake High-Tech Developing Zone, Wuhan, Hubei, China 430079, catalog number: GB113808). NF-κB analyzed at dilution of 1:200 (from ABclonal, 500W Cummings Park, Ste. 6500 Woburn, MA 01801, United States, catalog number: A2547).

Image J software was used to enter the high-resolution photos of the whole tissue sections. To determine the relative antigen concentration, a virtual dissection or the extraction of the ROI (region of interest) in entire tissue sections at magnifications up to 40× were performed. On a microscope slide, the brown DAB chromogen is separated from the hematoxylin counterstain by processing the digital image using the color deconvolution method. The area fraction of DAB (antigen) was then calculated after a monochrome image containing the DAB content was submitted to frequency analysis using ImageJ Software (FIJI, National Institutes of Health, USA). The results are shown as means ± SEM and as a percentage of the immunopositive stained area in each pulmonary segment (Helps et al. 2012).

### Real-time quantitative polymerase Chain reaction (qPCR)

Gene expression of lung CTR1 and LOX was assessed by `PCR. Total RNA was extracted from tissue homogenate using SV Total RNA Isolation system (Thermo Scientific, USA). The total RNA (1μg) was used for cDNA conversion using high capacity cDNA reverse transcription kit (#K4374966, Thermo Fisher Scientific, USA). Real-time qPCR amplification and analysis were performed using an Applied Biosystem with software version 3.1 (StepOne™, USA). Thermocycler Rotor-Gene Q and SYBR Green PCR Master Mix were used to reverse-transcribe the RNA and run the PCR reaction in triplicate. The qPCR assay with the primer sets: (F: 5′-TTGGCTTTAAGAATGTGGACCT-3′ and R: 5′-CAT AAG GAT GGTTCCATTTGGT-3′ for CTR1, F: 5′-GCATACAGGGCAGATGTCAGA-3′ and R: 5′-TTGGCATCAAGCAGGTCATAG-3′ for LOX, and F: 5'CTA CGT CGC CCT GGA CTT CGA GC3′ and R: 5′GAT GGA GCC GCC GAT CCA CAC GG3 for β-actin as housekeeping gene were optimized at the annealing temperature. After the qPCR run the data were expressed in cycle threshold (Ct) and he target genes were calculated via ΔΔCt method.

### Nuclear extract preparation

As directed by the manufacturer, we worked with a Nuclear Extraction Kit (Novus Biologicals, Colorado, USA, Catalog number: NBP2-29,447) to separate nuclear proteins. In short, tiny pieces of lung tissue were homogenized in 5 ml of 1× Hypotonic Buffer supplemented with 1 mM DTT and 1% detergent, rinsed twice with 5 ml of ice-cold PBS/PMSF buffer, and then placed on ice for 30 min. Following a 10-min centrifugation at 10,000 rpm at 4 °C, the supernatants (cytoplasmic fraction) were extracted, and 500 µl of Complete Lysis Buffer was added to the nuclei. They were then rocked for 30 min at 4 °C. After vortexing, the samples were centrifuged for 10 min at 4 °C at 14,000 rpm. Nuclear protein-containing supernatants were collected and put into tubes that had already been cooled. The internal control was histone (Song et al. 2007).

### Western blot analysis

Based on (Harlow & Lane, 1999) method protein expression of CTR1, LOX, NF-κB and IκB was determined by Western blotting in accordance with the guidelines provided by the relevant manufacturer (CTR1 kits from FabGennix, 9191 Kyser Way, Bldg. 4 Suite 400, Frisco, TX 75033, USA, catalog number: CTR1-101AP & LOX (Catalog number: PA1-16953), NF-κB (Catalog number:14-6731-81), IκB (Catalog number: MA5-16152), Nrf2 (Catalog number: PA5-27882), Histone (Catalog number: PA5-16183) and β-Actin (Catalog number: MA1-140) kits from Invitrogen, Thermo Fisher Scientific, 168 Third Avenue Waltham, MA, USA 02451). In brief, protease and phosphatase inhibitors were added to 5 mL of RIBA lysis solution, which was used to homogenize the lungs on ice. After centrifuging homogenates for 30 min at 4 °C at ~ 16,000xg g, the supernatant was kept at -80°C until it was needed. The Bradford Protein Assay Kit (SK3041) was used to quantitatively measure the total protein concentrations. Before loading on polyacrylamide gel electrophoresis, 5–20 ug of protein from each sample was loaded with an equal volume of 2× Laemmli sample buffer. The mixture was then boiled for 5 min at 95 °C to guarantee that the protein was denaturated. The process, known as SDS-PAGE (for Sodium Dodecyl Sulfate Polyacrylamide Gel Electrophoresis), involved separating proteins on a polyacrylamide gel. Acrylamide and N, N-methylene bis-acrylamide (Bis is a cross-linking agent for the gel) were the two compounds that were polymerized to create polyacrylamide gels, which were left to polymerize for 30 to 45 min prior to electrophoresis. Tetramethylethylenediamine (TEMED) and ammonium per sulfate were added to start the polymerization process. To determine the protein size and track the development of an electrophoresis run, a molecular weight marker (BLUelf pre dyed protein ladder, GeneDireX, Taiwan, Cat No. PM008-0500) was utilized. To enable sample migration in the stacking layer, the gel was operated at 50 V for 20 min. In order to complete the run in roughly one hour, the voltage was raised to 100–150 V to permit protein movement and separation in the resolving layer. The membrane was incubated with primary antibodies against CTR1, LOX, NF-κB, Nrf2 and IκB (1:1000) diluted in RIBA buffered saline pH = 7.4 (TBS) with 0.1% Tween-20, per the manufacturer’s instructions, after being blocked for one hour at room temperature in tris-buffered saline with Tween 20 (TEST) buffer and 3% bovine serum albumin (BSA). Following TBS-Tween washing, membranes were incubated for one hour at room temperature with goat anti-rabbit IgG HRP-linked secondary antibody, and proteins were detected by enhanced chemiluminescence. ImageJ software was used to quantify the signals.

### Enzyme-linked immunosorbent assays (ELISAs)

The levels of tumor necrosis factor-alpha (TNF-α), interleukin (IL)-6, transforming growth factor-beta (TGF-β), phosphorylated SMAD3 (pSMAD3), collagen type I alpha 1 (COL1A1), CTR1, and LOX were measured in accordance with the guidelines provided by the relevant manufacturer. (TNF-α kits from CUSABIO, 7505 Fannin St., Ste 610, Room 7 (CUBIO Innovation Center), Houston, TX 77054, USA, Catalog number CSB E11987r & IL-6 kits from Quantikine® ELISA, Minneapolis, MN 55413, USA, Catalog number R6000B; TGF-β kits from MyBioSource, Inc. San Diego, USA, Catalog number MBS260302; SMAD3 kits from MyBioSource, Inc. San Diego, USA, Catalog number MBS269938; COL1A1 kits from Biomatik, Biomatik Corporation (head office), 4 Third Ave, Kitchener, Ontario, N2C 1N6, Canada, catalog number EKU03302 96 Tests; CTR1 kits from LSBIO, Vector Laboratories, Inc. 6737 Mowry Ave, Newark, CA 94560, United States, catalog number LS-F33681 and LOX kits from MyBioSource, Inc. San Diego, USA, catalog number MBS2514886).

### Statistical analysis

Graphpad Prism V8.0 Software (San Diego, CA, USA) was used for statistical analysis and graphing. Data were expressed as mean ± the standard error of the mean (SEM). One-way analysis of variance (ANOVA) followed by Tukey-Kramers multiple comparisons post hoc test were used for parametric measurements. Otherwise, the Kruskal–Wallis tests followed by the Dunn post hoc test were applied for non-parametric measures. At *p* < 0.5, statistical significance was deemed acceptable.

## Results

### Effect of trientine on lung injury biomarkers in bleomycin induced pulmonary fibrosis

**Fig. 1:**
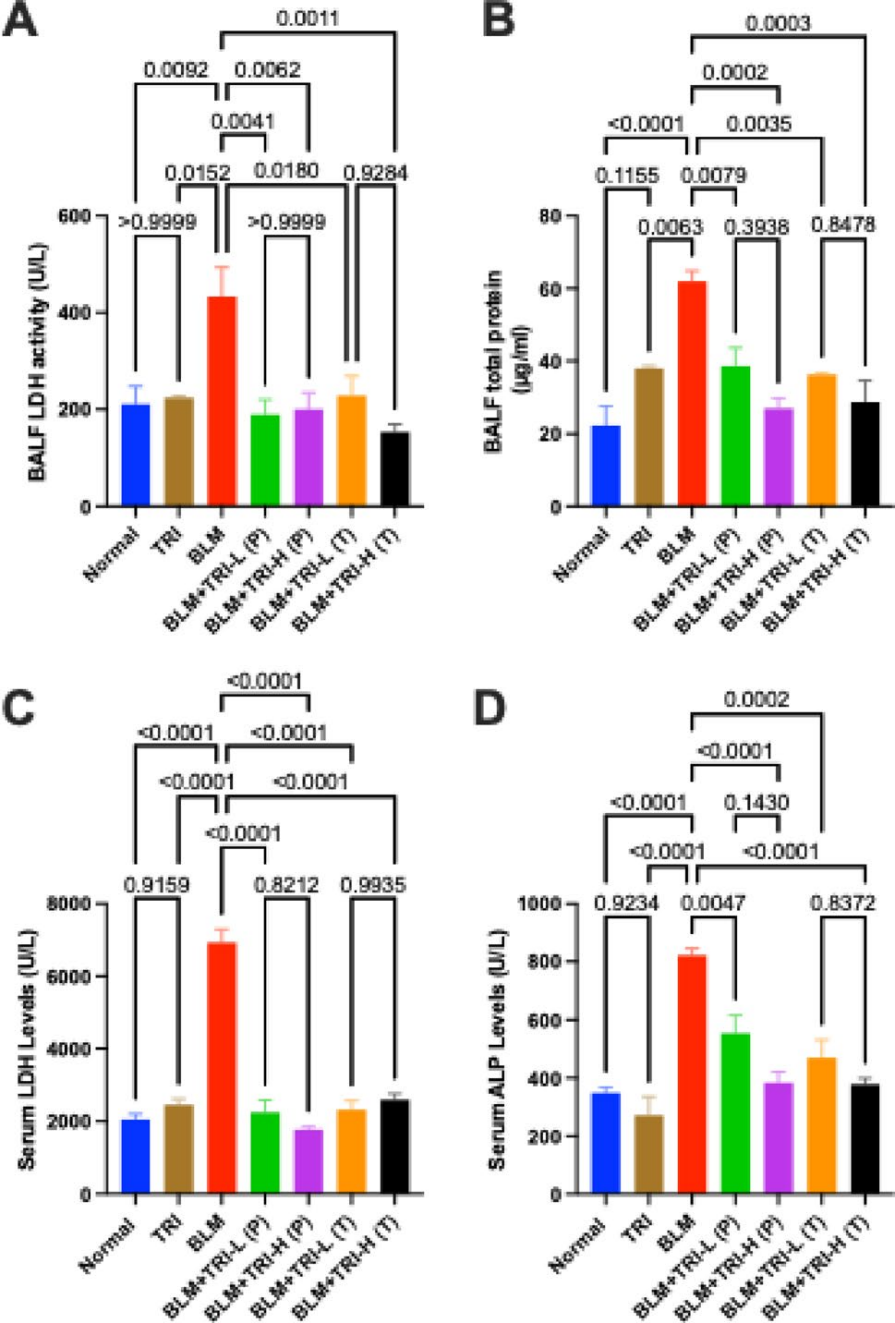
Effect of trientine on levels of BALF LDH (**A**), BLAF total protein (**B**), serum LDH (**C**), and serum ALP (**D**). The data is presented as mean ± SEM, n = 8. One-way ANOVA was used to statistically evaluate the data, and then the Tukey–Kramer multiple comparison test was used. *P* ≤ 0.05 indicates a statistical significance. TRI; Trientine group: rats received Trientine (110 mg/kg/day, oral) dissolved in 0.9% normal saline daily, BLM; Bleomycin control group: rats received bleomycin intratracheally at a dose of 5 mg/kg, BLM + TRI-L (P); Group pre-treated with 55 mg/kg day 3 days before BLM administration, BLM + TRI-H (P); Group pre-treated with 110 mg/kg day 3 days before BLM administration, BLM + TRI-L (T); Group treated with 55 mg/kg day after BLM administration, BLM + TRI-H (T); Group treated with 110 mg/kg day after BLM administration

LDH and total protein levels in the BALF were significantly higher in the BLM group than in the normal group. When compared to the BLM group, prophylactic or therapeutic intervention with TRI was associated with reductions in levels of total protein and LDH. Moreover, our data revealed that serum levels of LDH and ALP were considerably higher in BLM group than the normal group. Similarly, TRI was associated with lower serum LDH and ALP levels compared to the BLM group (Fig. 1).

### Effect of trientine on lung redox status in bleomycin induced pulmonary fibrosis

**Fig. 2:**
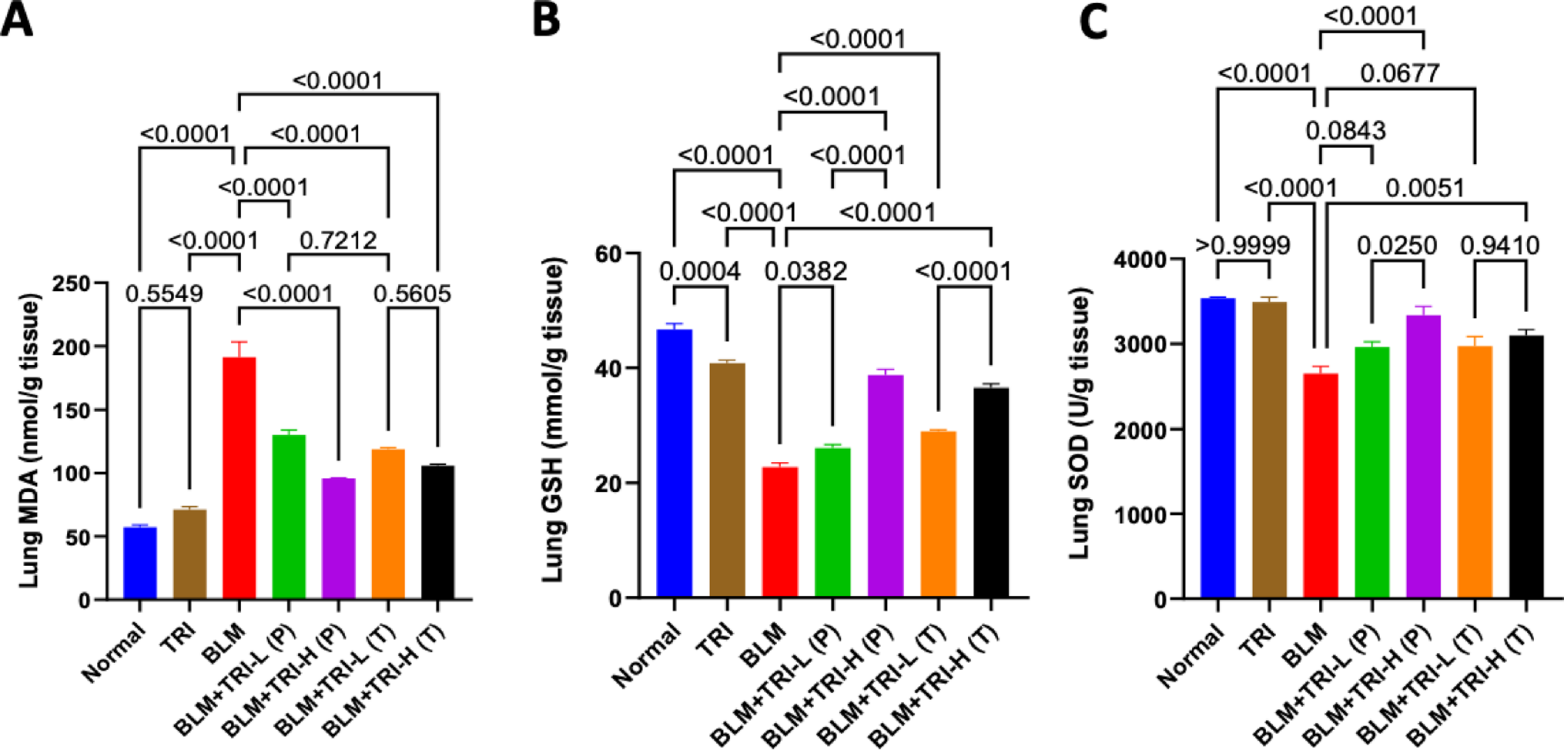
Effect of trientine on oxidative stress markers where: lung MDA (**A**), lung GSH (**B**) and lung SOD (**C**). The data is presented as mean ± SEM, n = 8. One-way ANOVA was used to statistically evaluate the data, and then the Tukey–Kramer multiple comparison test was used. *P* ≤ 0.05 indicates a statistical significance. TRI; Trientine group: rats received Trientine (110 mg/kg/day, oral) dissolved in 0.9% normal saline daily, BLM; Bleomycin control group: rats received bleomycin intratracheally at a dose of 5 mg/kg, BLM + TRI-L (P); Group pre-treated with 55 mg/kg day 3 days before BLM administration, BLM + TRI-H (P); Group pre-treated with 110 mg/kg day 3 days before BLM administration, BLM + TRI-L (T); Group treated with 55 mg/kg day after BLM administration, BLM + TRI-H (T); Group treated with 110 mg/kg day after BLM administration

Compared to normal group, intratracheal instillation of BLM resulted in a considerable rise in lung MDA content and a significant decrease in lung GSH content and SOD activity. Meanwhile, TRI prophylactically and therapeutically appeared to attenuate these changes in a dose-related manner compared to the BLM group (Fig. 2).

### Effect of trientine on histopathological inflammatory changes in bleomycin induced pulmonary fibrosis

**Fig. 3:**
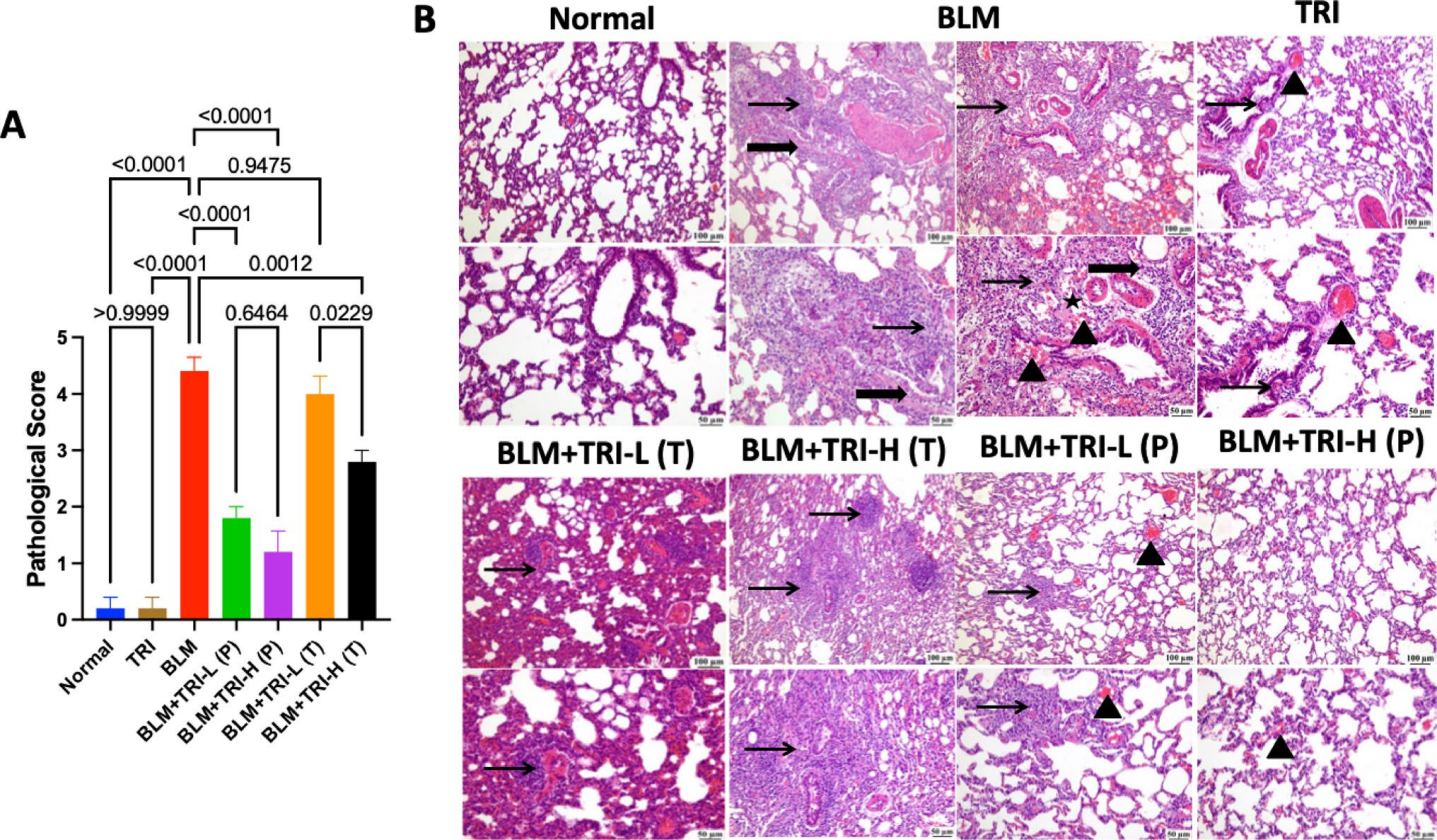
Representative photomicrograph of lung tissue sections from different treatment groups. Normal lung (low and high power) showing normal histological appearance of alveolar and bronchiolar architecture. Bleomycin showing diffuse interstitial pneumonia characterized by severe cellular infiltrates composed of abundant lymphoplasmacytic cells, macrophages admixed with mild edema, hemorrhage and severe fibrosis replacing and surrounding bronchiolar epithelial necrosis. Group treated by trientine 110 mg showing normal architecture of up to 85% of pulmonary tissue except few focal intraluminal bronchiolar mononuclear inflammatory cells and mild vascular congestion. Group treated by trientine 55 mg showing few intrabronchilar desquamated epithelial cells with multifocal interstitial inflammatory aggregates mostly composed of lymphocytes and mild numbers of macrophages and fibroblasts. Group treated by trientine 110 mg showing multifocal to coalescing extensive peribronchiolar inflammation admixed with moderate fibrosis replacing and compressing the remaining alveoli. Group protected with trientine 55 mg showing focal interstitial inflammatory nodules composed of centerally necrotic centers surrounded by many lymphocytes, plasma cells and macrophages together with abundant fibroblasts. Group protected with trientine 110 mg showing normal architecture of up to 85–90% with few interalveolar hemorrhage. Thin arrow = inflammation, thick arrow = fibrosis, arrowhead = congestion or hemorrhage, star = edema. Image magnification = 100×, 400×. The data is presented as mean ± SEM. Kruskal–Wallis test was used to statistically evaluate the data, followed by Dunn’s multiple comparison test. *P* ≤ 0.05 indicates a statistical significance. TRI; Trientine group: rats received Trientine (110 mg/kg/day, oral) dissolved in 0.9% normal saline daily, BLM; Bleomycin control group: rats received bleomycin intratracheally at a dose of 5 mg/kg, BLM + TRI-L (P); Group pre-treated with 55 mg/kg day 3 days before BLM administration, BLM + TRI-H (P); Group pre-treated with 110 mg/kg day 3 days before BLM administration, BLM + TRI-L (T); Group treated with 55 mg/kg day after BLM administration, BLM + TRI-H (T); Group treated with 110 mg/kg day after BLM administration

Upon H&E staining, the optimum lung architecture was found in rats of the normal group and rats in the TRI group were less or more comparable with the normal rats. On the other hand, the BLM group showed a malformed lung structure, showing clear and noticeable thickening of the interalveolar septa due to a persistent inflammatory cellular infiltration which is represented in a higher pathological score. In comparison to the BLM group, pre- or post-treatment with TRI demonstrated improved histological appearance and lower the pathological score compared to the BLM group in a dose-related manner (Fig. 3).

It is noteworthy to mention that the TRI group did not have any significant effect on lung injury biomarkers, lung redox status, lung histopathological inflammatory changes when compared to the normal group. Additionally, the previous results indicated that the prophylactic effect of TRI appeared somewhat more effective than its therapeutic effect, so we completed this study with TRI as a prophylactic treatment to investigate the potential underlying mechanism of action.

### Effect of trientine on expression of the nuclear factor erythroid 2-related factor 2 in bleomycin induced pulmonary fibrosis

**Fig. 4:**
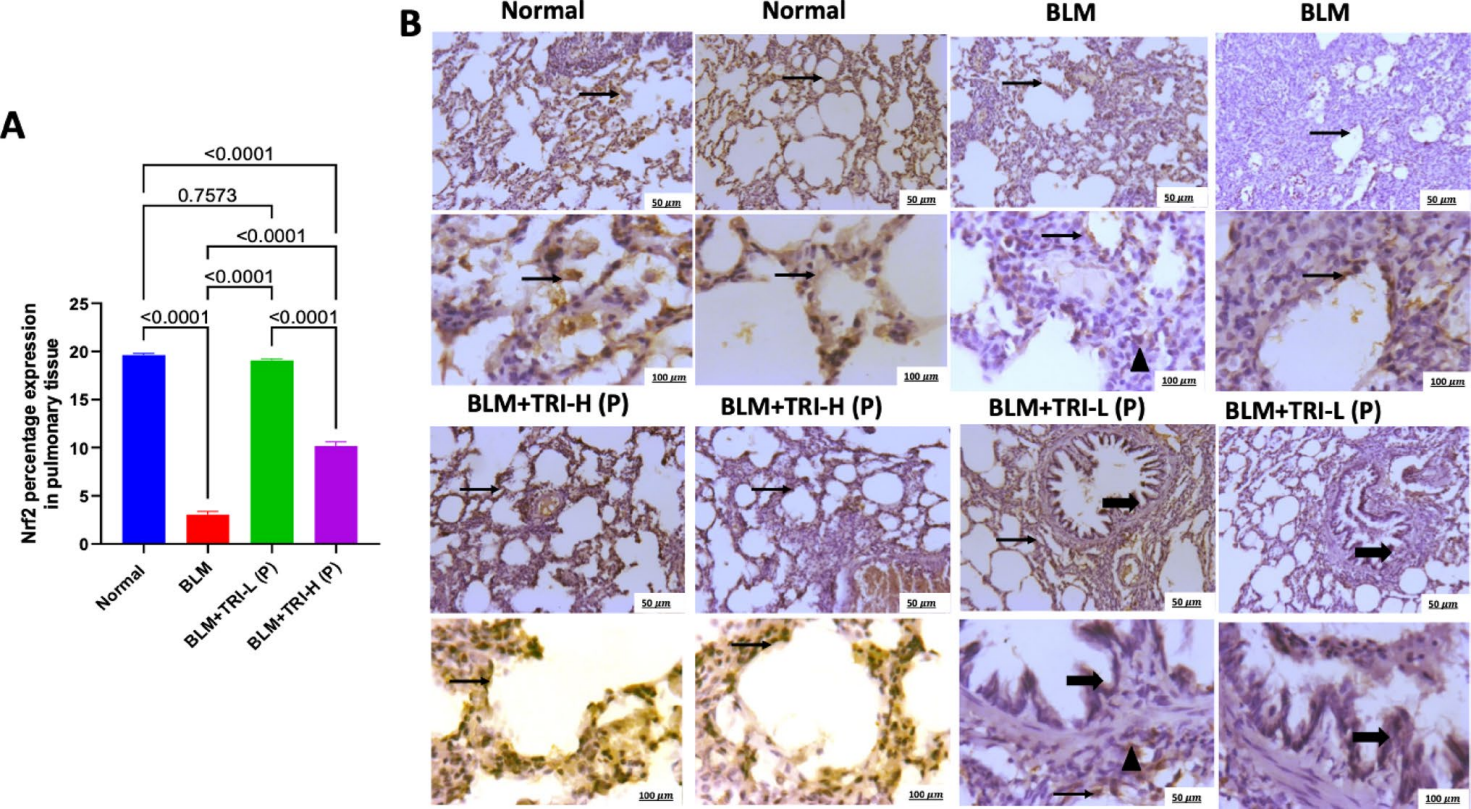
Effect of trientine on levels of Nrf2 in lung tissue (**A**) and Representative IHC of Nrf2 from pulmonary sections of different treatment groups (**B**). The data is presented as mean ± SEM. One-way ANOVA was used to statistically evaluate the data, and then the Tukey–Kramer multiple comparison test was used. P ≤ 0.05 indicates a statistical significance. Normal group showing moderate to high immunostained alveolar epithelial cells and interstitial cells. Bleomycin group showing few immunopositivity in alveolar epithelial cells and intestitial inflammatory cells. Group protected with trientine 110 mg showing moderate to high immunopositive stained bronchiolar and alveolar epithelial cells. Group protected with trientine 55 mg showing mild to moderate immunopositive stained bronchiolar and alveolar epithelium with mild expression in interstitial inflammatory cells. Thin arrow = positive alveolar epithelial cells, thick arrow = positive bronchiolar epithelial cells, arrowhead = positive interstitial cells. Image magnification = 100×, 400×. BLM; Bleomycin control group: rats received bleomycin intratracheally at a dose of 5 mg/kg, BLM + TRI-L (P), BLM + TRI-L (P); Group pre-treated with 55 mg/kg day 3 days before BLM administration, BLM + TRI-H (P); Group pre-treated with 110 mg/kg day 3 days before BLM administration, BLM + TRI-L (T); Group treated with 55 mg/kg day after BLM administration, BLM + TRI-H (T); Group treated with 110 mg/kg day after BLM administration

**Fig. 5:**
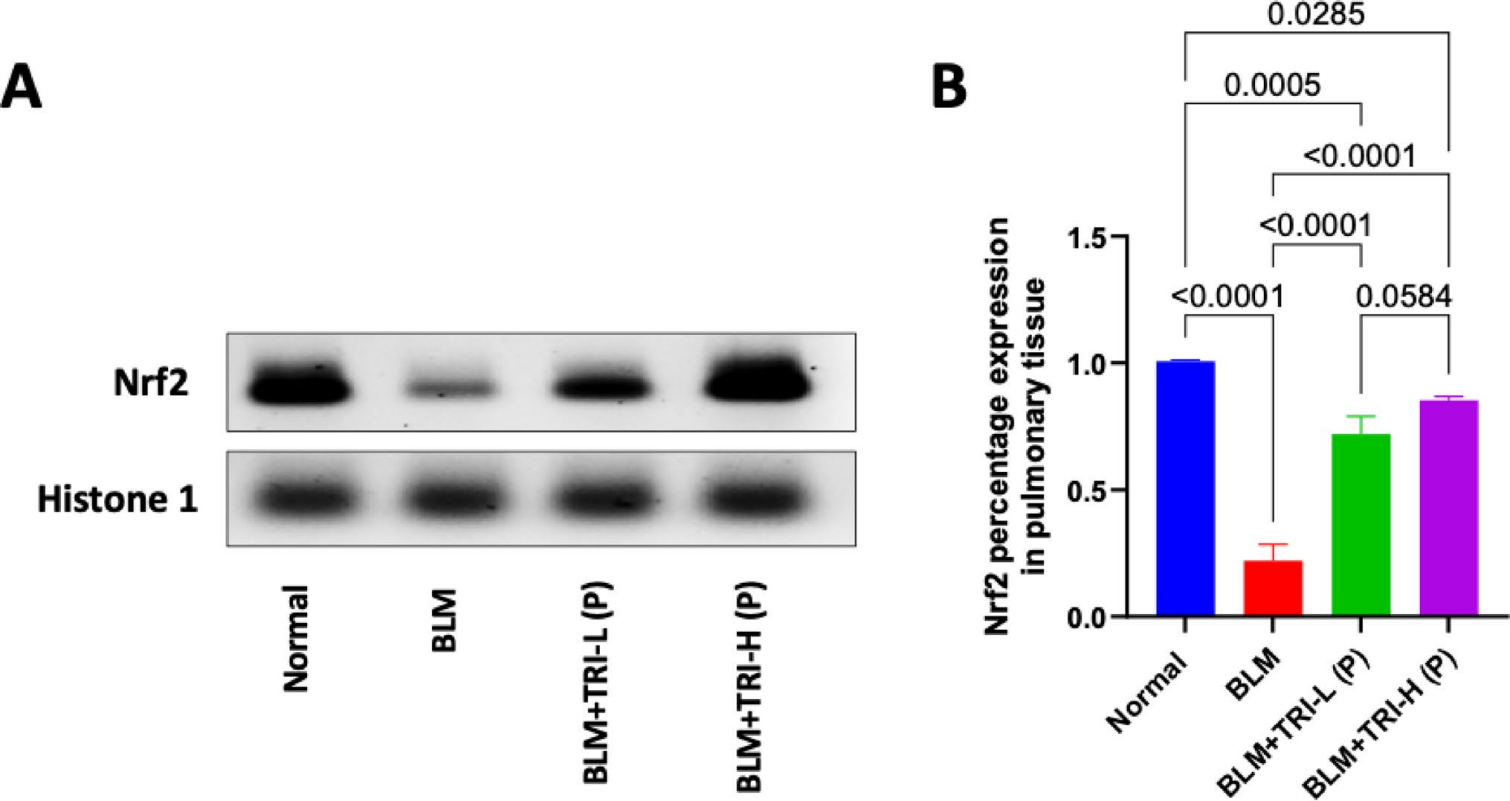
Effect of trientine on protein expression of Nrf2 in lung tissue, **A** Representative western blots of histone and Nrf2 proteins for different groups, **B** Nrf2 expression levels in the lung. The data is presented as mean ± SEM. One-way ANOVA was used to statistically evaluate the data, and then the Tukey–Kramer multiple comparison test was used. *P* ≤ 0.05 indicates a statistical significance. BLM; Bleomycin control group: rats received bleomycin intratracheally at a dose of 5 mg/kg, BLM + TRI-L (P), BLM + TRI-L (P); Group pre-treated with 55 mg/kg day 3 days before BLM administration, BLM + TRI-H (P); Group pre-treated with 110 mg/kg day 3 days before BLM administration, BLM + TRI-L (T); Group treated with 55 mg/kg day after BLM administration, BLM + TRI-H (T); Group treated with 110 mg/kg day after BLM administration

As shown in Fig. 4, the normal group showing moderate to high Nrf2 immunostained alveolar epithelial cells and interstitial cells. On the other hand, the BLM group showing few immunopositivity in alveolar epithelial cells and intestitial inflammatory cells. Meanwhile, pre-treatment with low-dose TRI showing mild to moderate immunopositive stained bronchiolar and alveolar epithelium with mild expression in interstitial inflammatory cells. Otherwise, pre-treatment with high-dose TRI showing moderate to high immunopositive stained bronchiolar and alveolar epithelial cells. These results were represented by the percentage Nrf2 expression. Our findings revealed that BLM dramatically reduced protein expression of lung Nrf2 in comparison to the normal group. Meanwhile, pre-treatment with TRI significantly increased Nrf2 expression levels in the lung, showing a dose-related manner (Fig. 5).

### Effect of trientine on ceruloplasmin, copper transporter 1, and lysyl oxidase enzymes in bleomycin induced pulmonary fibrosis

**Fig. 6:**
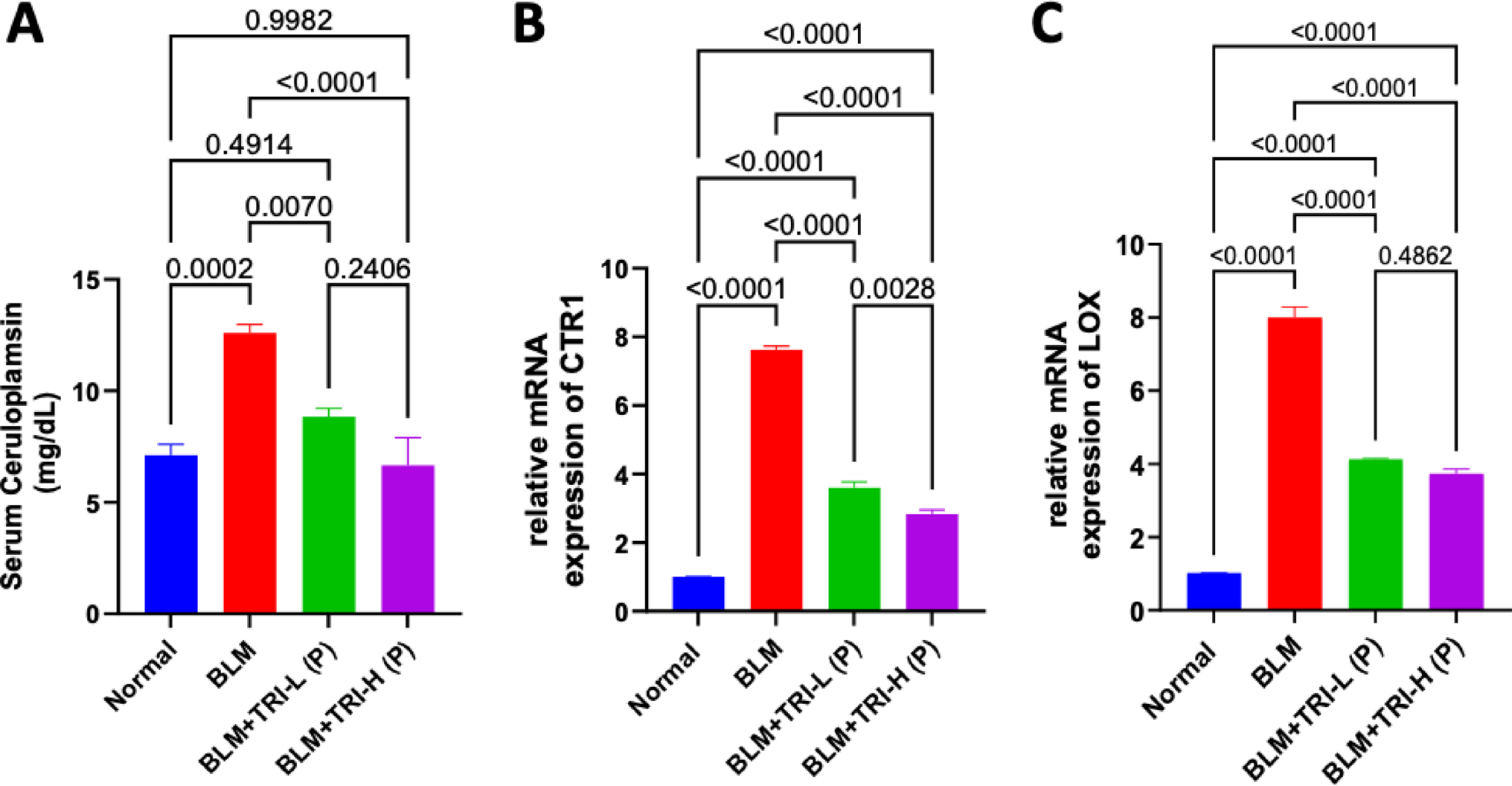
Effect of trientine on levels of seum ceruloplasmin (**A**), relative mRNA expression of CTR1 (**B**) and LOX (**C**) in lung tissue. The data is presented as mean ± SEM, n = 8. One-way ANOVA was used to statistically evaluate the data, and then the Tukey–Kramer multiple comparison test was used. *P* ≤ 0.05 indicates a statistical significance. BLM; Bleomycin control group: rats received bleomycin intratracheally at a dose of 5 mg/kg, BLM + TRI-L (P), BLM + TRI-L (P); Group pre-treated with 55 mg/kg day 3 days before BLM administration, BLM + TRI-H (P); Group pre-treated with 110 mg/kg day 3 days before BLM administration, BLM + TRI-L (T); Group treated with 55 mg/kg day after BLM administration, BLM + TRI-H (T); Group treated with 110 mg/kg day after BLM administration

**Fig. 7:**
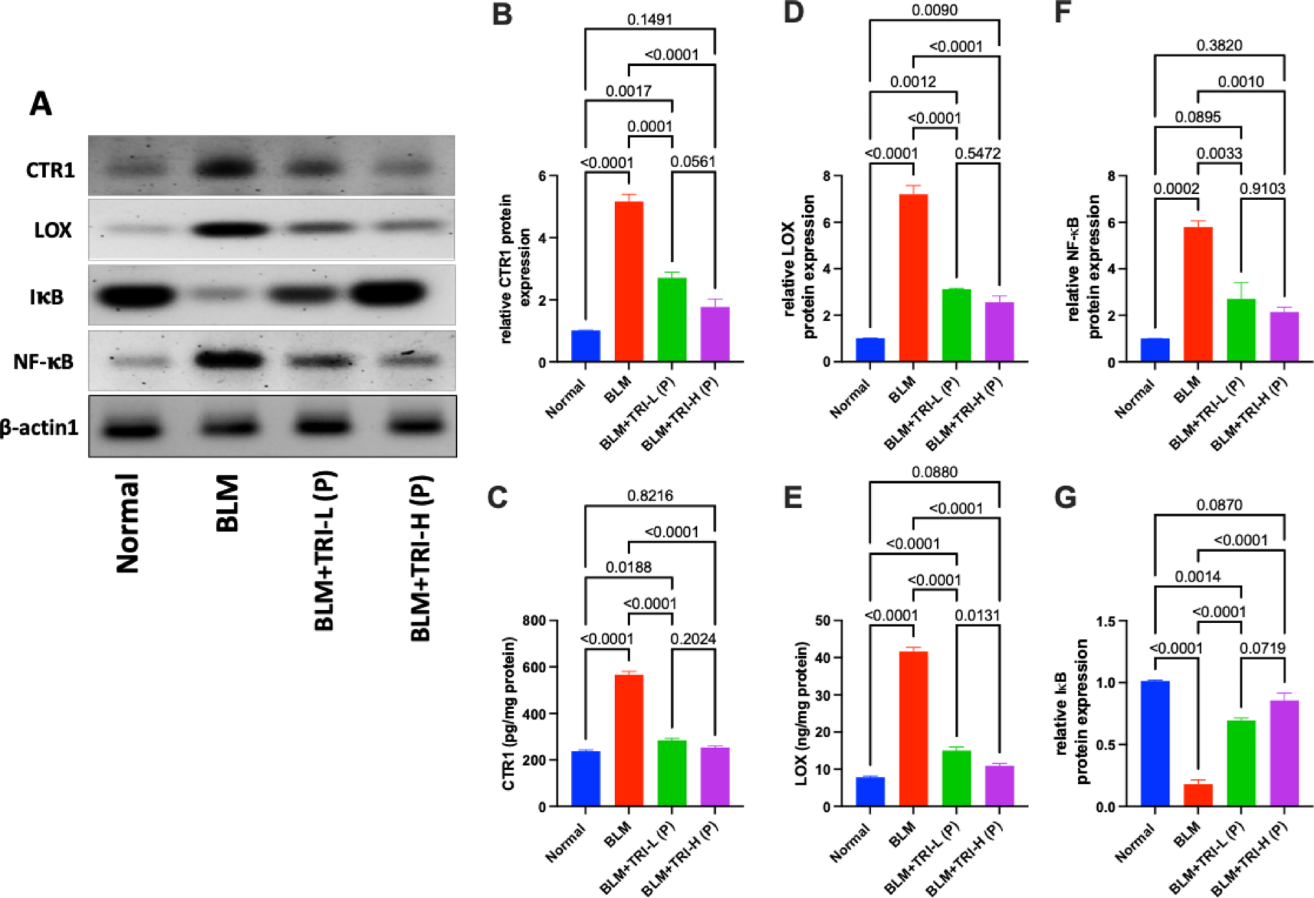
Effect of trientine on the expression of CTR1, LOX, NF-κB and IκB proteins. **A** Representative western blots of β-actin, CTR1, LOX, NF-κB and IκB proteins for different groups. **B, C** Expressions of CTR1 measured by WB and ELISA respectively. **D**, **E** Expression of LOX measured by WB and ELISA respectively. **F** Expression of NF-κB measured by WB. **G** Expression of IκB measured by WB.The data is presented as mean ± SEM, n = 3 and 8 respectively. One-way ANOVA was used to statistically evaluate the data, and then the Tukey–Kramer multiple comparison test was used. *P* ≤ 0.05 indicates a statistical significance. BLM; Bleomycin control group: rats received bleomycin intratracheally at a dose of 5 mg/kg, BLM + TRI-L (P), BLM + TRI-L (P); Group pre-treated with 55 mg/kg day 3 days before BLM administration, BLM + TRI-H (P); Group pre-treated with 110 mg/kg day 3 days before BLM administration, BLM + TRI-L (T); Group treated with 55 mg/kg day after BLM administration, BLM + TRI-H (T); Group treated with 110 mg/kg day after BLM administration

As showed in Fig. 6, Serum levels of ceruloplasmin were considerably higher in bleomycin group than the normal group. When compared to the BLM group, TRI was associated with lower serum ceruloplasmin levels. Regarding CTR1 and LOX, our findings revealed that BLM dramatically increased gene and protein expression of lung CTR1 and LOX in comparison to the normal group. Meanwhile, pre-treatment with TRI was associated with reductions in CTR1 and LOX expression levels in the lung in a dose-related manner (Figs. 6 and 7).

### Effect of trientine on nuclear factor kappa B and inhibitor of nuclear factor kappa B in in bleomycin induced pulmonary fibrosis

**Fig. 8:**
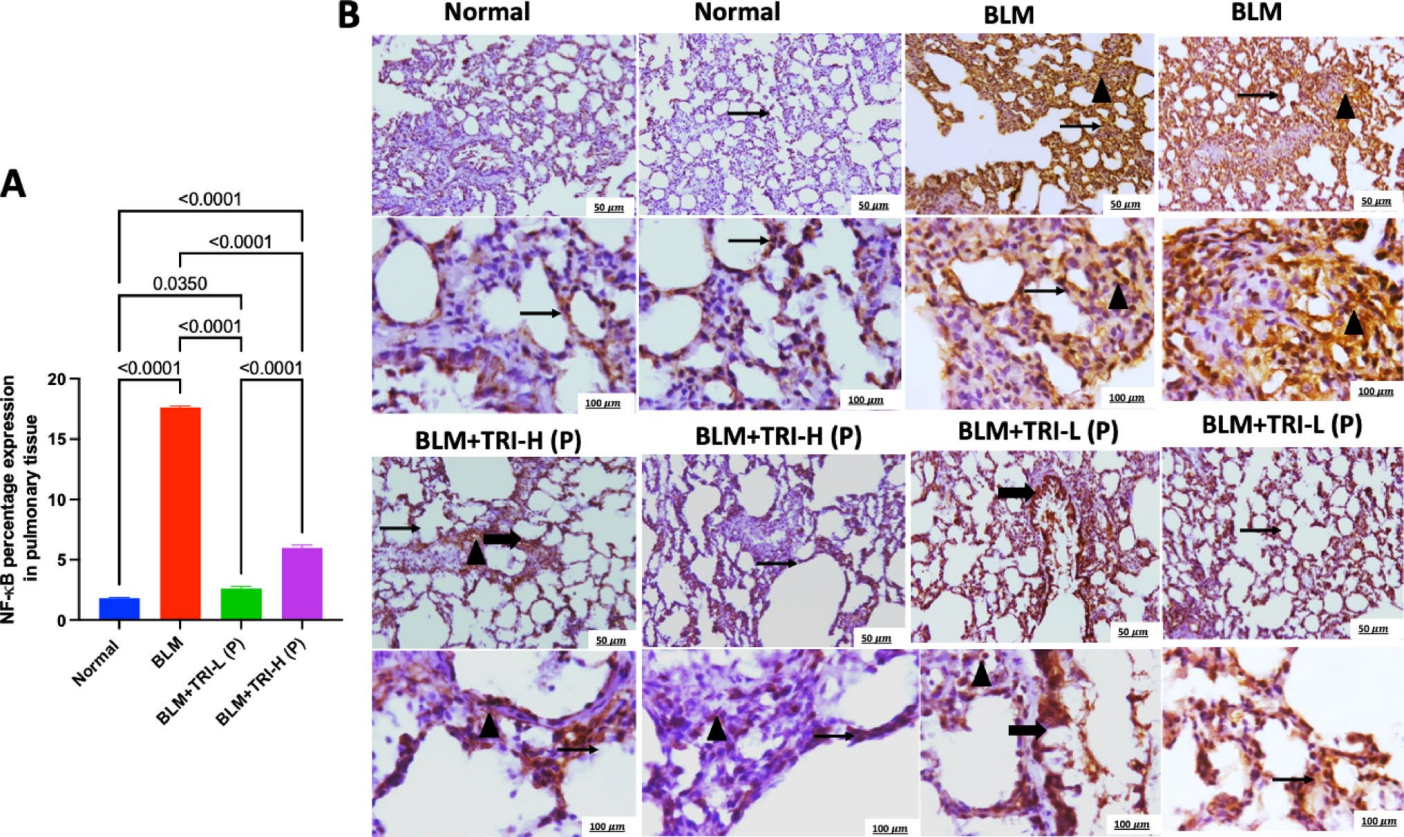
Effect of trientine on levels of NF-κB in lung tissue (**A**) and Representative IHC of NF-κB from pulmonary sections of different treatment groups (**B**). Normal group showing faint immunostained alveolar epithelial cells and interstitial cells. Bleomycin group showing diffuse high intense immunopositive stained interstitial and peribronchiolar inflammatory cells with immunopositivity in alveolarand bronchiolar epithelial cells. Group protected with trientine 110 mg showing mild immunopositive stained bronchiolar and alveolar epithelium with mild positivity in interstitial inflammatory cells. Group protected with trientine 55 mg showing mild to moderate immunopositive stained bronchiolar and alveolar epithelium with interstitial inflammatory cells nuclear positivity. Thin arrow = positive alveolar epithelial cells, thick arrow = positive bronchiolar epithelial cells, arrowhead = positive interstitial cells. Image magnification = 100×, 400×. The data is presented as mean ± SEM. One-way ANOVA was used to statistically evaluate the data, and then the Tukey–Kramer multiple comparison test was used. *P* ≤ 0.05 indicates a statistical significance. BLM; Bleomycin control group: rats received bleomycin intratracheally at a dose of 5 mg/kg, BLM + TRI-L (P), BLM + TRI-L (P); Group pre-treated with 55 mg/kg day 3 days before BLM administration, BLM + TRI-H (P); Group pre-treated with 110 mg/kg day 3 days before BLM administration, BLM + TRI-L (T); Group treated with 55 mg/kg day after BLM administration, BLM + TRI-H (T); Group treated with 110 mg/kg day after BLM administration

As showed in Fig. 8, the BLM group has diffuse high intense NF-κB immunopositive staining interstitial and peribronchiolar inflammatory cells with immunopositivity in alveolarand bronchiolar epithelial cells compared to faint immunostained alveolar epithelial cells and interstitial cells in the normal group. Meanwhile, the group protected with low-dose TRI has mild to moderate immunopositive stained bronchiolar and alveolar epithelium with interstitial inflammatory cells nuclear positivity. Otherwise, the group protected with high-dose TRI has reveals immunopositive stained bronchiolar and alveolar epithelium with mild positivity in interstitial inflammatory cells. These results were represented by the percentage NF-κB expression.

Additionally, NF-κB and IκB protein expression was addressed by Western blotting. Our data revealed that the BLM group has significantly higher NF-κB expression while lower IκB expression compared to the normal group. Meanwhile, TRI administration was associated with partial prevention of these changes.

### Effect of trientine on tumor necrosis factor-alpha and interleukin-6 in bleomycin induced pulmonary fibrosis

**Fig. 9:**
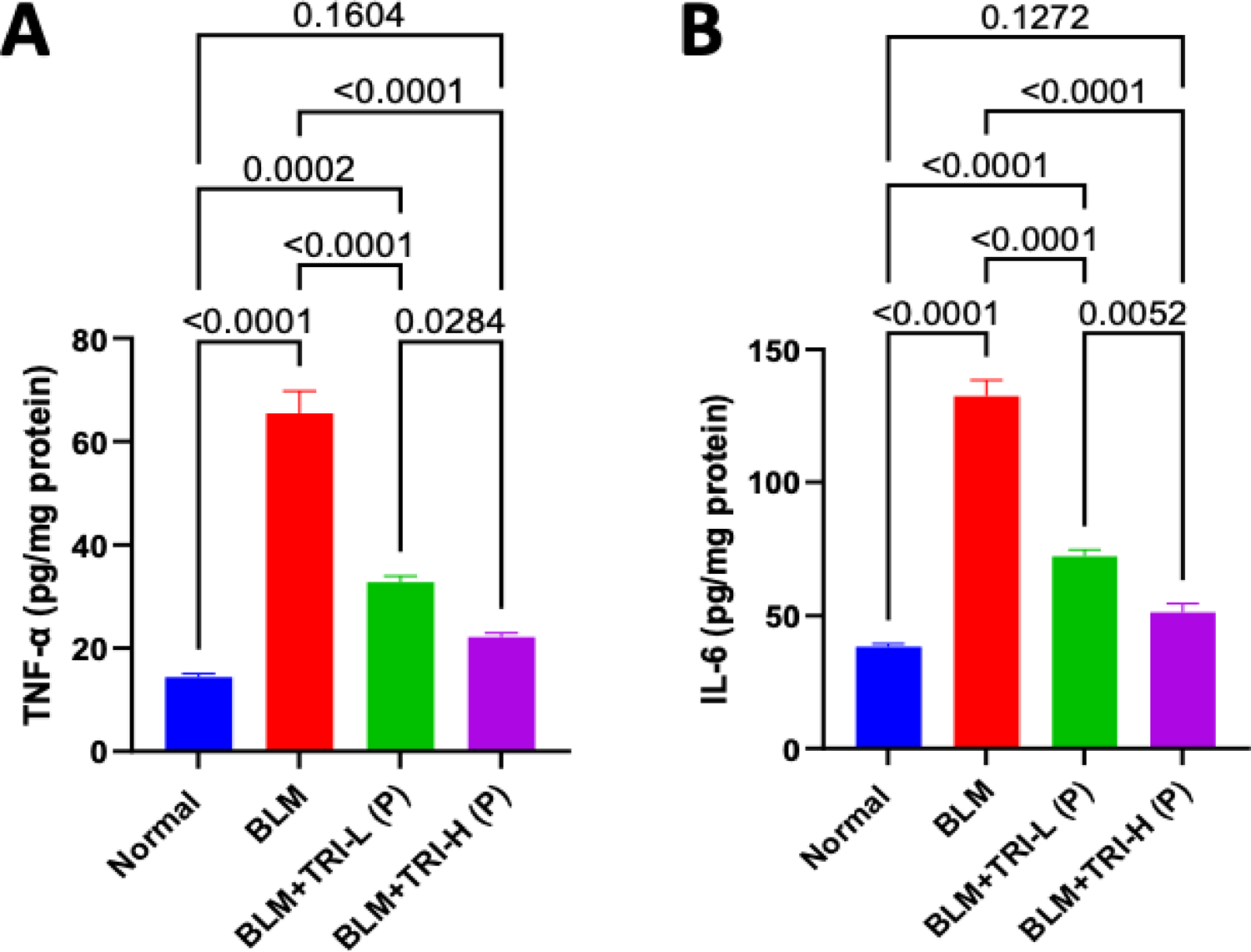
Effect of trientine on levels of: TNF-α and IL-6 in lung tissue. The data is presented as mean ± SEM, n = 8. One-way ANOVA was used to statistically evaluate the data, and then the Tukey–Kramer multiple comparison test was used. *P* ≤ 0.05 indicates a statistical significance. BLM; Bleomycin control group: rats received bleomycin intratracheally at a dose of 5 mg/kg, BLM + TRI-L (P), BLM + TRI-L (P); Group pre-treated with 55 mg/kg day 3 days before BLM administration, BLM + TRI-H (P); Group pre-treated with 110 mg/kg day 3 days before BLM administration, BLM + TRI-L (T); Group treated with 55 mg/kg day after BLM administration, BLM + TRI-H (T); Group treated with 110 mg/kg day after BLM administration

When BLM was administered, the lung TNF-α and IL-6 levels significantly increased in comparison to the normal group. When compared to the BLM group, TRI administration was associated with lower TNF-α and IL-6 levels in the lung, showing a dose-related effect (Fig. 9).

### Effect of trientine on histopathological fibrotic changes in bleomycin induced pulmonary fibrosis

**Fig. 10:**
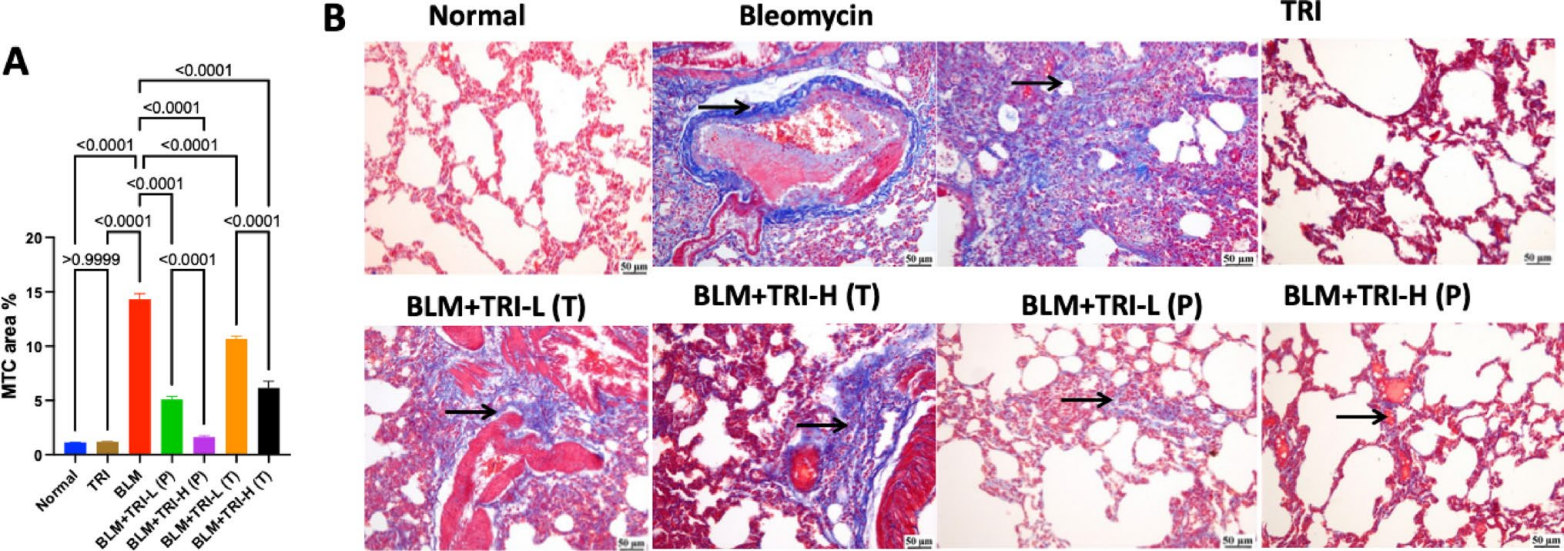
Representative Masson’s trichrome micrograph of lung from different treatment groups. Normal group showing normal alveolar architecture. Bleomycin showing high dense bluish peribronchiolar and interstitial collagen deposition. Trienine (110 mg) group showing negative to few peribronchiolar tiny fibrous connective tissue. Group treated by trientine 55 mg showing moderate bluish interstitial fibrosis. Group treated by trientine 110 mg showing mild focal peripronchiolar bluish fibrous connective tissue or scattered faint bluish stained collagen deposition. Group protected with trientine 55 mg showing minimal intestitial bluish stained fibrous connective tissue. Group protected by trientine 110 mg showing few peribronchial bluish stained fibrous connective tissue. Image magnification = 400×, scale bar = 50 µm. The data is presented as mean ± SEM. One-way ANOVA was used to statistically evaluate the data, and then the Tukey–Kramer multiple comparison test was used. P ≤ 0.05 indicates a statistical significance. TRI; Trientine group: rats received Trientine (110 mg/kg/day, oral) dissolved in 0.9% normal saline daily, BLM; Bleomycin control group: rats received bleomycin intratracheally at a dose of 5 mg/kg, BLM + TRI-L (P); Group pre-treated with 55 mg/kg day 3 days before BLM administration, BLM + TRI-H (P); Group pre-treated with 110 mg/kg day 3 days before BLM administration, BLM + TRI-L (T); Group treated with 55 mg/kg day after BLM administration, BLM + TRI-H (T); Group treated with 110 mg/kg day after BLM administration

Masson’s Trichome staining was used to evaluate the fibrotic changes in the lung tissue. Rats in the normal group showed no fibrosis and a normal lung histological appearance, In contrast, the BLM group revealed a significant development of ECM components, which raised the fibrotic score. Conversely, the prophylactic and therapeutic TRI groups reduced fibrotic alterations and lowered the fibrosis score, especially the prophylactic groups (Fig. 10).

### Effect of trientine on transforming growth factor beta, phosphorylated SMAD3 and Collagen type I in bleomycin induced pulmonary fibrosis

**Fig. 11:**
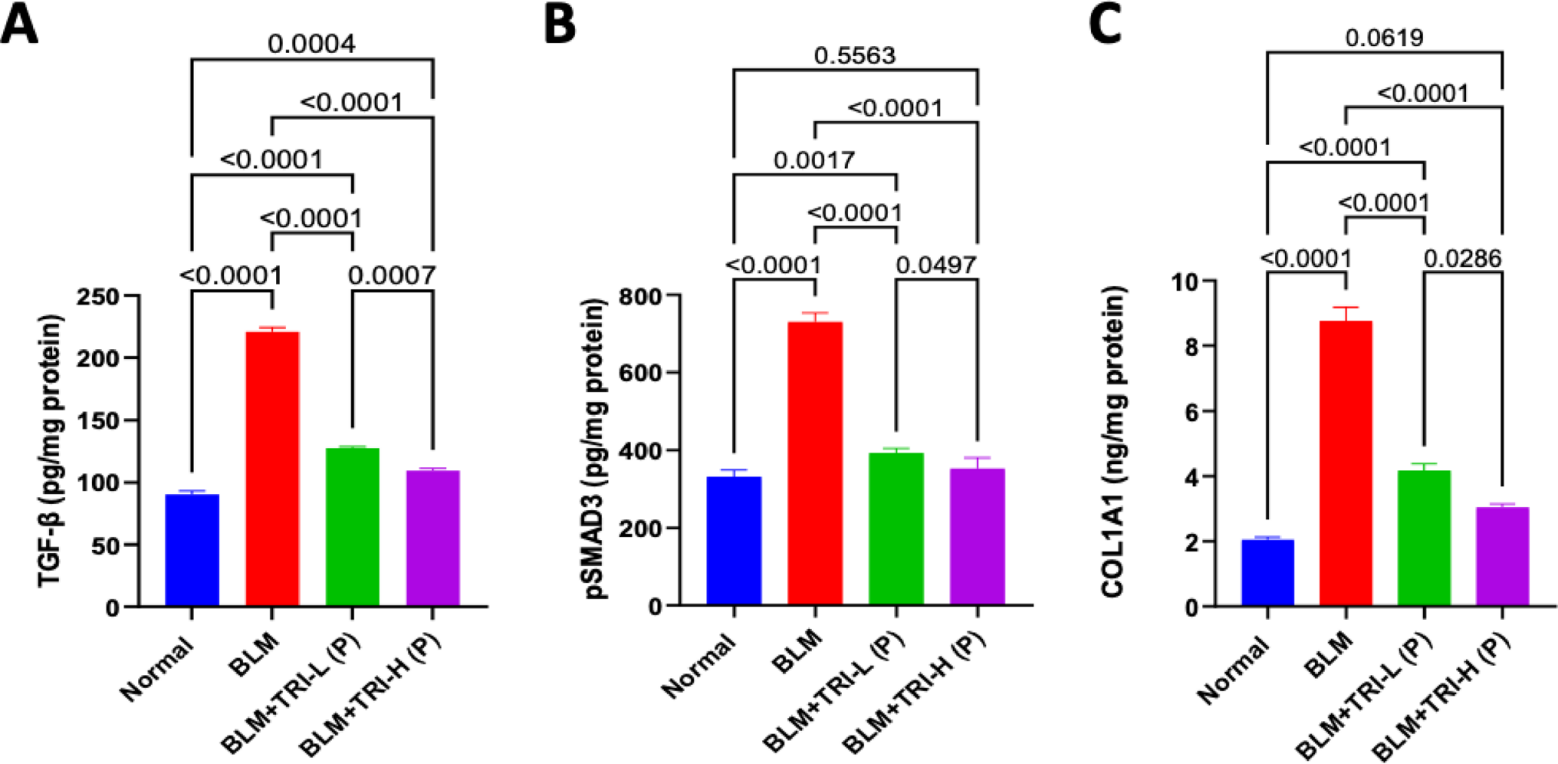
Effect of trientine on levels of TGF-β (**A**), pSMAD3 (**B**) and COL1A1 (**C**). The data is presented as mean ± SEM, n = 8. One-way ANOVA was used to statistically evaluate the data, and then the Tukey–Kramer multiple comparison test was used. *P* ≤ 0.05 indicates a statistical significance. BLM; Bleomycin control group: rats received bleomycin intratracheally at a dose of 5 mg/kg, BLM + TRI-L (P), BLM + TRI-L (P); Group pre-treated with 55 mg/kg day 3 days before BLM administration, BLM + TRI-H (P); Group pre-treated with 110 mg/kg day 3 days before BLM administration, BLM + TRI-L (T); Group treated with 55 mg/kg day after BLM administration, BLM + TRI-H (T); Group treated with 110 mg/kg day after BLM administration

When BLM was administered TGF-β, pSMAD3, and COL1A1 levels in the lung significantly increased in comparison to the normal group. When compared to the BLM group, TRI administration, in a dose-related manner, was associated with reductions in TGF-β, pSMAD3, and COL1A1 in the lung tissues (Fig. 11).

## Discussion

In the present study, intra-tracheal administration of BLM resulted in a significant increase in the BALF’s total protein levels and LDH activity. It also resulted in a significant increase in serum LDH and ALP levels. These results are consistent with earlier research that found that in the BLM-induced IPF model, there was an increase in total protein (indicating pulmonary edema), LDH (indicating cytotoxicity), and ALP (indicating inflammation) (Zaghloul et al. 2019). It’s interesting to note that TRI reduced the amount of total protein in BALF, which diminished the intensity of lung and alveolar inflammation. Furthermore, TRI administration significantly reduced serum ALP and also LDH activity in both BALF and serum.

The two most important characteristics of lung fibrosis are oxidative stress and inflammation. It has been demonstrated that BLM causes production of reactive oxygen species (ROS) and oxidative stress in the lung. In the current examination of the antioxidant defense mechanisms (SOD and GSH) activities, BLM increased the amount of MDA in the lung while decreasing SOD and GSH (Pan et al. 2023; Zhang et al. 2025). Fortunately after TRI administration, MDA levels are decreased, while SOD and GSH activities significantly increased, demonstrating TRI’s antioxidant activities.

These findings are consistent with earlier studies, although little is known about TRI’s effects on inflammation and oxidative stress. It has been shown to alleviate oxidative stress and pathological diseases in the liver (Seetharaman and Sarma 2021) and the heart (Liu et al. 2018; Ramli et al. 2022; Farrant et al. 2023). TRI has been shown to enhance SOD expression in experimental diabetic cardiomyopathy models (Zhang et al. 2013, which is crucial for the cells’ protection against oxidative stress.

The main function of the transcription factor Nrf2 is to control the antioxidant responses of cells (Vomund et al. 2017). The Nrf2-antioxidant signaling pathway is triggered in response to oxidative stress, which promotes the transcription of several antioxidant defense enzymes, such as SOD (Ngo and Duennwald 2022). Downstream of Nrf2 and its antioxidant enzymes have been shown to play important roles in the pathophysiology of PF (Wang et al. 2022).

In the present study, the BLM-injected rats showed downregulated Nrf2 protein expression when compared to the normal control rats. This finding is consistent with earlier research showing that BLM may lower Nrf2 expression (El-Horany et al. 2023; Li et al. 2024), whereas TRI intervention successfully upregulated Nrf2 expression.

Ceruloplasmin, also known as Cu oxidase, is a glycoprotein manufactured in the liver, and it carries more than 90% of Cu in serum; therefore, we can regard ceruloplasmin as the storage form of Cu in the blood (Zulkufli et al. 2022). It has been linked to pulmonary fibrosis and was discovered to be markedly increased in the serum of patients with silicosis (Beshir et al. 2015). As an acute-phase response protein, ceruloplasmin is also markedly increased in both concentration and enzymatic activity in response to infection, inflammation, and trauma (Vasilyev 2010; Squitti et al. 2019). In our research BLM increased ceruloplasmin levels, and similar findings from earlier studies supported the present findings (Derseh et al. 2021). Administration of TRI decreased these elevated ceruloplasmin levels.

Pulmonary fibrosis is characterized by the accumulation of large amounts of ECM ingredients, especially collagen and elastin, which are stabilized by Cu (Wang et al 2024). About 70% of the uptake of Cu in human cells is attributed to CTR1, which is distinguished by its high affinity for Cu (Hasan and Lutsenko 2012). It’s noteworthy to note that elevated Cu concentrations have been noticed in the BALF of PF patients (Song et al. 2024), and also Cu-dependent enzymes, including LOX, are involved in the pathophysiology of lung fibrosis (Chen et al. 2018). The production of the cupric-dependent enzyme LOX and the crosslinking of the pro-elastin polymer seem to be the most crucial of the several elastin repair processes (Kothapalli and Ramamurthi 2009). According to Janssen et al. 2018, the local availability of Cu to activate enough LOX for elastin crosslinking is what distinguishes PF from emphysema (Janssen et al. 2018). As TRI is a Cu chelator, we investigated TRI’s possible ability to inhibit the Cur-mediated CTR1/LOX/COL pathway both before and after BLM induction. To the best of our knowledge, this study is the first to investigate the effect of TRI on IPF via modulating the CTR1/LOX/COL pathway. Our results showed that BLM significantly increased levels of serum ceruloplasmin, the expression of CTR1, LOX, and COL1 whereas TRI successfully reduced their expression in protected and treated groups. This suggests that TRI may have inhibitory activity against the Cu/CTR1/LOX/COL pathway and can reduce the PF that BLM induces.

Pro-inflammatory cytokines have been shown to have a major role in the pathophysiology of BLM-induced PF (She et al. 2021; Mohammed et al. 2024). The generation of ECM, collagen deposition, and fibroblast proliferation can be induced by cytokines (Younesi et al. 2024). BLM has been shown to induce the release of pro-inflammatory cytokines (Ayilya et al. 2023) and NF-κB, which controls the transcription of certain cytokine genes, such as TNF-α and IL-6 (El-Bassouny et al. 2021). The enzyme complex known as IκB suppresses NF-κB and maintains its dormant state in the cytoplasm (Thakur et al. 2022). In the current study, TRI dramatically reduced the increased levels of proinflammatory cytokines (TNF-α and IL-6) and NF-κB activity while it elevated IκB levels in BLM-injected rats.

Another important growth factor that mediates the pathophysiology of lung fibrosis is the profibrogenic TGF-β1, which elicits signals that interact with Smad mediators so fibroblasts can produce a lot of collagen (Ye and Hu 2021). Smad 2/3 is activated by TGF-β1, and Smad4 then binds to the active Smad2/3. This complex translocates to the nucleus. Myofibroblasts are activated, and ECM is deposited as a result of the complex’s Smad 2/3 component directly attaching to gene promoters to initiate the transcription of profibrotic molecules such as COLI and α-SMA (Hata and Chen 2016; Pal et al. 2022; An et al. 2024). The impact of TRI on the TGF-β1/Smad signaling pathway has been examined in rats with diabetic cardiomyopathy and showed an inhibitory effect on the expression of the SMAD gene and left ventricular TGF-β1, which was linked to decreases in collagen (Lu et al. 2013; Ramli et al. 2022). Herein, TRI significantly suppressed the TGF-β1 expression in the lung of BLM-injected rats. Increased extracellular Cu levels facilitate the accumulation of advanced glycated end products in collagen, which in turn encourages TGF-β1 induced collagen deposition (Cui et al. 2022). Consequently, TRI’s reduction of Cu probably inhibits collagen accumulation and also lowers the production of TGF-β1. All the aforementioned results are compatible with our histological findings that revealed suppressed inflammatory and fibrotic changes in the TRI-treated rats.

## Conclusion

The present study suggests that the Cu chelator, TRI may exert protective effects against BLM-induced pulmonary fibrosis in rats by modulating oxidative stress, inflammation, and fibrotic pathways. TRI treatment was associated with reductions in serum ceruloplasmin levels and CTR-1 protein expression therefore, a reduction in the production of LOX enzymes. Importantly, TRI maintains IκBα expression, thereby preventing NF-κB activation, and also inhibit the TGF-β1/Smad3 pathway. These molecular changes were accompanied by improved regulation of pro-inflammatory (TNF-α and IL-6) and oxido-inflammatory (Nrf2) indicators, which may help reduce collagen deposition in PF. As a result, TRI therapy decreases the PF progression. While these findings are promising, they are based on an animal model, and further studies, including dose–response evaluations and clinical trials, are needed to confirm TRI’s safety and therapeutic potential in humans.

## Limitations

This study has several important limitations. First, it was conducted in a BLM-induced pulmonary fibrosis rat model, which may not fully replicate the complexity of idiopathic pulmonary fibrosis in humans, limiting direct clinical applicability. Second, TRI was evaluated using a single-dose design and fixed treatment duration, without exploring dose–response relationships or long-term effects. Third, functional assessments such as pulmonary function tests were not performed, which are essential for correlating molecular findings with physiological outcomes. Additionally, TRI was not compared with standard antifibrotic therapies like pirfenidone or nintedanib, restricting conclusions about its relative efficacy. Finally, as the findings are preclinical, further studies, including well-designed clinical trials, are necessary to confirm TRI’s safety, pharmacokinetics, and therapeutic potential in humans.

## Data Availability

No datasets were generated or analysed during the current study.

## Abbreviations

PF: Pulmonary fibrosis
ECM: Extracellular matrix
Cu: Copper
CTR1: Copper transporter
LOX: Lysyl oxidase
TRI: Trientine tetrahydrochloride
BLM: Bleomycin
BALF: Bronchoalveolar lavage fluid
LDH: Lactate dehydrogenase
ALP: Alkaline phosphatase
ROS: Reactive oxygen species
SOD: Superoxide dismutase
GSH: Reduced glutathione
MDA: Malondialdehyde
TGF-β: Transforming growth factor-beta
COL 1: Collagen type 1
NRF2: Nuclear factor erythroid 2-related factor 2
TNF-α: Tumor necrosis factor
IL-6: Interleukin 6
NF-κB: Nuclear factor kappa B
IκB: Inhibitor of kappa B
BLM + TRI-L (P): Group pre-treated with 55 mg/kg day 3 days before BLM administration
BLM + TRI-H (P): Group pre-treated with 110 mg/kg day 3 days before BLM administration
BLM + TRI-L (T): Group treated with 55 mg/kg day after BLM administration
BLM + TRI-H (T): Group treated with 110 mg/kg day after BLM administration
H&E: Hematoxylin & eosin
qPCR: Quantitative polymerase Chain reaction
ELISA: Enzyme-linked immunosorbent assay
